# Increasing the activity of copper exchanged mordenite in the direct isothermal conversion of methane to methanol by Pt and Pd doping†
†Electronic supplementary information (ESI) available. See DOI: 10.1039/c8sc02795a


**DOI:** 10.1039/c8sc02795a

**Published:** 2018-10-03

**Authors:** P. Tomkins, A. Mansouri, V. L. Sushkevich, L. I. van der Wal, S. E. Bozbag, F. Krumeich, M. Ranocchiari, J. A. van Bokhoven

**Affiliations:** a Paul Scherrer Institut , CH-5232 Villigen , Switzerland; b ETH Zurich , Wolfgang-Pauli-Strasse 10 , CH-8093 Zurich , Switzerland . Email: jeroen.vanbokhoven@chem.ethz.ch; c Utrecht University , Universiteitsweg 99 , 3584 CG Utrecht , The Netherlands

## Abstract

PtCu- and PdCu–mordenite allow for isothermal reaction at 200 °C for the stepwise methane to methanol conversion with comparably high yields. In contrast to traditional Cu-zeolites, these materials are more reactive under isothermal conditions than after high temperature activation.

## Abstract 2

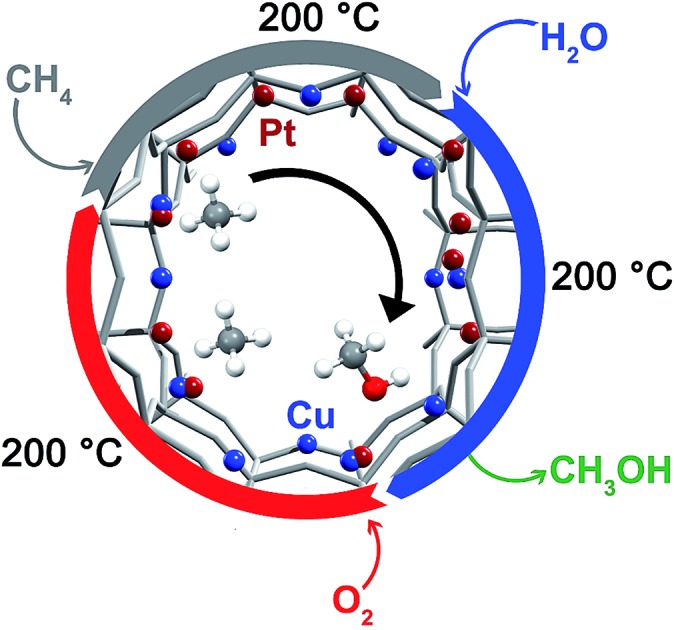
PtCu– and PdCu–mordenite allow for isothermal reaction at 200 °C for the stepwise methane to methanol conversion with higher yields under isothermal conditions than after high temperature activation.

## 

Methane is an abundant feedstock that can be extracted inexpensively from shale gas and other sources.^1^ Costs of transportation are comparably high and a major part of the methane, recovered as a side-product in oil fields, is flared.^1^ There are strategies for converting methane to higher value-added chemicals,^2^,^3^ but the conversion of methane to liquids is a complex process. Industrial methanol synthesis is energy intense, as it requires the production of synthesis gas. Methane products of partial oxidation are more reactive than methane itself,^3^ which fundamentally limits the yield of methanol from the direct methane oxidation.^4^–^6^ Successes have been achieved on systems that produce derivatives of initial oxidation products, however, these turned out not to currently be industrially feasible.^7^,^8^

Methane monooxygenase enzymes transform methane to methanol at low temperature with active dicopper^9^ or diiron^10^ sites. The same motif has also been transferred to solid catalysts based on Fe-,^11^–^13^ Cu-14–23 and Co-exchanged^24^,^25^ zeolites, silica and alumina. High temperature (≥450 °C) activates these materials by means of molecular oxygen.^22^ The activated material then reacts with methane at a significantly lower temperature (50 °C to 200 °C), forming strongly adsorbed methanol precursors. The adsorbed species can be extracted off-line with a liquid or on-line in a wet gas stream (Fig. S1†).^18^,^22^ Proposals for the structure of the active sites are bis-μ-oxo,^14^,^17^ mono-μ-oxo dicopper sites^16^ and most recently tris-oxo sites.^15^,^18^,^23^,^26^,^27^ Brønsted acid sites showed to be beneficial for the reaction^28^ and the reactivity matches well with that expected for a Cu^I^/Cu^II^ redox couple.^29^ Copper-loaded small-pore zeolites are also active.^22^ For Cu-CHA a mono-Cu site was proposed as possible active site or as precursor to the active site.^30^ Generally, oxygen is used during activation and the active sites start being formed at about 280 °C in Cu-ZSM-5 (ref. 15) and, generally, activation above 450 °C is beneficial.^31^ We most recently investigated the reaction, in which water can be used as oxidant after activation at high temperature.^32^,^33^ Maximum yields achieved so far were 160 μmol g^–1^ for Cu-MOR and 125 μmol g^–1^ for Cu-CHA.^23^,^34^ Most reports focus on a single stoichiometric conversion, but reusability has already been proven.^18^,^35^ Isothermal cycles with nitrogen monoxide as the oxidant resulted in very low methanol yields of up to 0.629 μmol g^–1^, assumed to be achieved over the mono-μ-oxo site.^21^ We recently discovered the isothermal stepwise conversion of methane to methanol at 200 °C using Cu-MOR with yields up to 56.2 μmol g^–1^.^36^ In this case the activation in oxygen, reaction with methane and extraction with water can be performed successively at the same temperature (Fig. S1†). Several cycles of the reaction could be carried out with constant methanol yield. Higher methane pressures were employed to allow for the reaction to happen over the less reactive clusters, of which the stability generally increases with cluster size.^37^ Further, Cu-oxo clusters of different structure possess different activity under aerobic and anaerobic conditions.^38^ This approach showed that it is fundamentally possible to allow for isothermal reaction by changing the reaction parameters; we set out to search for novel materials that allow for higher activity at low temperature.

To date, most studies use monometallic copper-zeolites based on different topologies and material compositions for the transformation and these have so far not showed increased activity under isothermal conditions. Based on the well-known interplay between copper and platinum group metals,^39^ we envisioned mixed bimetallic Cu-oxo particles as being suitable for the low temperature oxidation of methane to methanol. Platinum group metals are often excellent C–H activation catalysts under mild conditions,^7^,^40^ which might aid the reactivity at low temperature. In addition, oxygen activation in mixed Cu–Pd and Cu–Pt complexes was recently described by Cramer and Tolman, which was proposed for direct methane to methanol conversion.^41^ This might not only result in a changed (re-)oxidation process of the material, but also lead to different reactivity towards methane. We herein investigate the stepwise conversion of methane to methanol using PtCu-MOR and PdCu-MOR materials. In contrast to all copper zeolites, a decreasing activity with increasing activation temperature was observed and yields under isothermal conditions at 200 °C were significantly higher than for Cu-MOR itself.

Cu-MOR_8.5_ with 4.3 wt% Cu was prepared by three-fold ion exchange of Na-MOR (Si/Al = 8.5) using Cu(OAc)_2_. A series of PtCu-MOR_8.5_(Pt/Cu_theory_) materials was prepared by suspending Cu-MOR_8.5_ in a solution containing the Pt(NH_3_)_4_(NO_3_)_2_ precursor, subsequently water was evaporated to yield the final material. Measured Pt and Cu contents can be found in Table S1.† Pt-content was lower than the employed amount of platinum. PtCu-MOR_8.5_(0.35) was prepared from a parent Cu-MOR_8.5_ with 2.4 wt% Cu. The Pt/Cu- and Pd/Cu-ratios were more thoroughly evaluated using samples with Si/Al = 6. Materials were prepared by impregnation of an analogously prepared Cu-MOR_6_. H_2_PtCl_6_ dissolved in water or PdCl_2_ dissolved in an aqueous HCl solution (30 wt%) were used, allowing for rather predictable loading of the platinum over a broad concentration range.

First, the effect of activation temperature on the methanol yield^18^ was investigated (Fig. 1). The material was activated in a flow of oxygen at the designated temperature, after which the reactor was maintained at to 200 °C. After purging the reactor with helium, methane was introduced at ambient pressure for 30 min and the reactor cooled down naturally in a flow of helium, after which the methanol was extracted off-line with liquid water. After activation at 200 °C, Cu-MOR_8.5_ yielded 0.8 μmol g^–1^. Note that after an isothermal reaction, Cu-MOR only releases MeOH during extraction with steam at 200 °C (Fig. S3†), but there are strongly adsorbed carbon-containing species remaining after extraction that are transformed to CO_2_ after heating in O_2_ at 300–350 °C (Fig. S4†). The final selectivity is approximately 93% in this case, which was determined by mass spectrometry of the extraction and subsequent oxidation of the strongly adsorbed species to CO_2_. The yield increased gradually after activation at 350 °C (8.7 μmol g^–1^) and at 450 °C (30.1 μmol g^–1^). The low activity of copper-zeolites at 200 °C activation and reaction is in agreement with the inactivity of Cu-ZSM-5 under similar conditions.^21^ Pt-MOR_6_ (1 wt% Pt) and Pd-MOR_6_ (2 wt% Pd) yielded 4.5 μmol g^–1^ and 2.0 μmol g^–1^ (Fig. 2), respectively. Both metals are known as C–H activation catalysts, where they are used as homogeneous Catalytica^7^ and Shilov systems.^42^

**Fig. 1:**
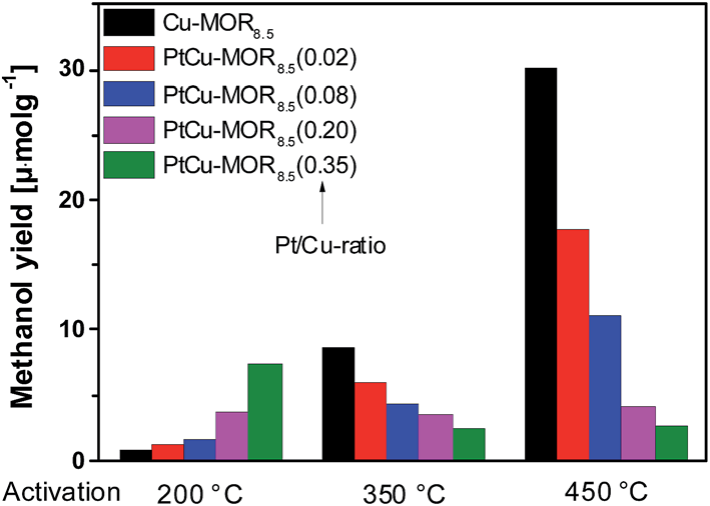
Methanol yields of different PtCu-MOR_8.5_(Pt/Cu_theory_) materials prepared from Cu-MOR (Si/Al = 8.5, 4.3 wt% Cu; ≤2.4 wt% for PtCu-MOR_8.5_(0.35)) after activation at different temperatures.

**Fig. 2:**
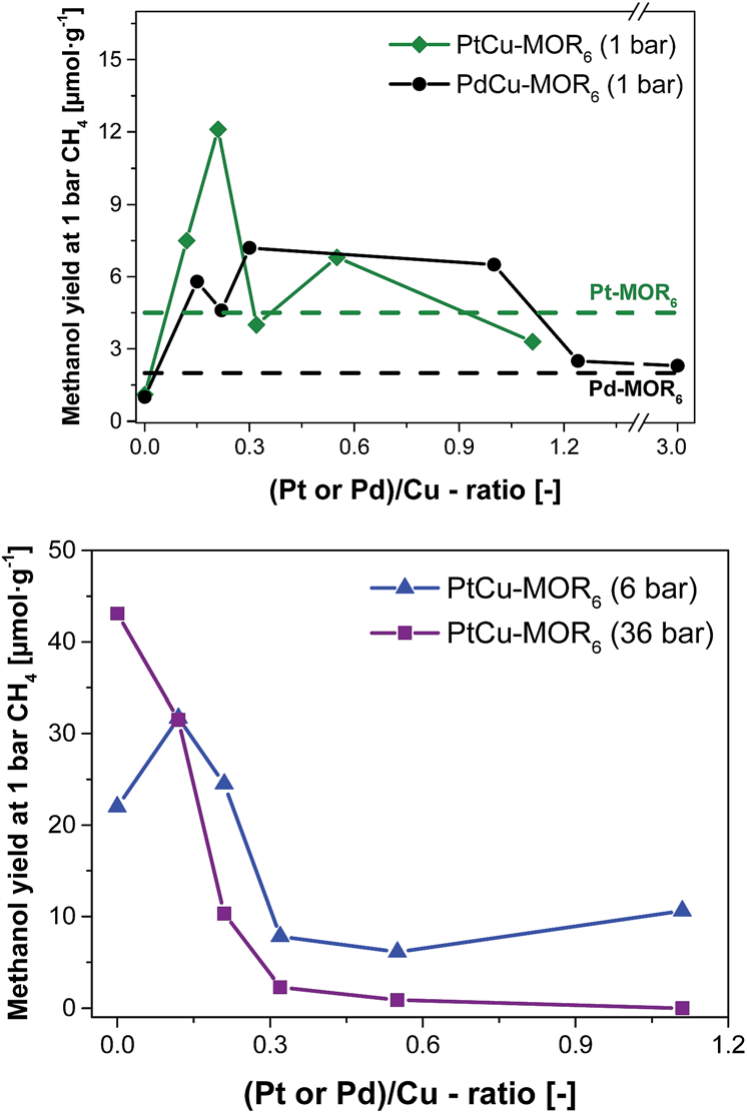
Methanol yield after activation in oxygen and reaction in methane at 200 °C with subsequent extraction in liquid water for PtCu-MOR_6_ and PdCu-MOR_6_ (Si/Al = 6) materials with different Pt/Cu- and Pd/Cu-ratios prepared by incipient wetness impregnation using H_2_PtCl_6_ and PdCl_2_ at ambient pressure (top) and elevated pressures (bottom). These yields are compared to the parent Pt-MOR and Pd-MOR. The yield for Cu-MOR_6_ at 6 bar CH_4_ was taken from literature.^36^

In analogy to the parent Cu-MOR_8.5_, the PtCu-MOR_8.5_ materials were employed in the cyclic methane to methanol conversion after activation at 200 °C, 350 °C and 450 °C (Fig. 1). Within this series there were two trends observable. On the one hand using low temperature activation the yield increased with increasing Pt/Cu-ratio from 0.8 μmol g^–1^ for Cu-MOR_8.5_ up to (7.4 μmol g^–1^) using PtCu-MOR_8.5_(0.35) under isothermal conditions at 200 °C. On the other hand, the yield decreased with increasing platinum content after 450 °C activation from 30.1 μmol g^–1^ for Cu-MOR_8.5_ to 2.7 μmol g^–1^ for PtCu-MOR_8.5_(0.35). At an intermediary activation temperature of 350 °C the platinum-rich zeolites were less active, but the effect is not as pronounced as observed at 450 °C activation. Thus, PtCu-MOR with increasing Pt/Cu-ratio provides higher activity under isothermal conditions, while the opposite is true after 450 °C activation. These results show the positive effect of platinum for the isothermal conversion. The beneficial effect of added platinum becomes even more obvious, when the yield is normalized to the amount of copper present in the material (Fig. S2†). Note that for materials that are doped with less Pt there is still an increase in yield with increasing activation temperature. This is caused by (rather large) fractions of the material being highly similar to parent Cu-MOR, which provide higher activity after high temperature activation. The beneficial effect of the added noble metal can stem from agglomeration of these noble metal precursors and the Cu-species to form bimetallic Cu-oxo clusters.

Next, the effect of Pt/Cu-ratio was studied at different methane pressures after activation in oxygen and reaction with methane at 200 °C followed by off-line extraction with liquid water to test the performance for the isothermal conversion (Fig. 2, elemental composition in Table S2†). Parent Cu-MOR_6_ gave a yield of 1.0 μmol g^–1^ at ambient CH_4_ pressure. With increasing Pt/Cu-ratio the yield increased to 12.5 μmol g^–1^ for PtCu-MOR(0.21), whereafter the yield decreased with increasing Pt/Cu-ratio to yield 3.3 μmol g^–1^ at Pt/Cu = 1.1. Thus, there is an optimal Pt/Cu-ratio providing maximum reactivity. At low Pt/Cu-ratios the effect of platinum introduction is low, while at higher Pt/Cu undesired agglomerates can be formed during activation or reaction. In analogy to Cu-MOR,^36^ higher methane pressures (6 and 36 bar) were tested in addition to investigate whether for this system higher yields can be achieved with higher methane pressures. There is a distinct difference to the reactions performed at 1 bar methane and those at higher pressures. At 6 bar, the parent Cu-MOR_6_ reached a yield of 22.0 μmol g^–1^, which further increased to 31.7 μmol g^–1^ for PtCu-MOR_6_(0.12), after which it decreased to minimum of 6.1 μmol g^–1^ for PtCu-MOR_6_(0.4). At 36 bar, the yield decreased monotonously from 43.1 μmol g^–1^ for the parent Cu-MOR_6_ until finally no methanol was extracted for PtCu-MOR_6_(1.1). These results indicate that an overall beneficial result can be obtained using higher methane pressures (*e.g.* for PtCu-MOR_6_(0.12)), yet increasing pressures and Pt-loading result in lower yields compared to the parent Cu-MOR_6_.

In the next step, palladium was investigated as the second metal, as it is known to activate C–H bonds and to undergo redox reactions with copper. A similar effect to PtCu-MOR was observed as palladium was introduced. Isothermal activation in oxygen and reaction in methane gave a maximum methanol yield of 7.2 μmol g^–1^ for PdCu-MOR_6_ (0.3). A slightly lower yield (6.5 μmol g^–1^) was obtained for PdCu-MOR(1.0), while Pd/Cu = 1.2 and 3.0 yielded ∼2.4 μmol g^–1^. The presence of Pd in PdCu-MOR_6_(0.3) was confirmed by EDX (Fig. S6†). This proves that the introduction of palladium improves the activity under isothermal conditions, making the approach of bimetallic oxide materials for the methane to methanol conversion more general. A mass spectrometric analysis of the reaction with PtCu-MOR_6_(0.15) and PdCu-MOR_6_(0.30) revealed the formation of a small amount of formaldehyde as only side-product, which is formed during the extraction of PdCu-MOR_6_(0.30) (Fig. S5†). The surface composition of PtCu-MOR_6_(0.15) and PdCu-MOR_6_(0.30) was compared after activation in oxygen using XPS. The noble metals are enriched on the surface with a Pt/Cu ratio of 0.5 and a Pd/Cu ratio of 0.7 (Table S3†). The ratio of PdCu-MOR_6_(0.30) is somewhat higher than that of PtCu-MOR_6_(0.15), which can be caused by the higher metal loading. Note that activation at 450 °C resulted in a lower yield of 20 μmol g^–1^ for PdCu-MOR_6_(1.0) compared to 27 μmol g^–1^ for the parent Cu-MOR_6_ under identical conditions (Table S4†). Thus, the introduction of platinum and palladium in Cu-MOR initially results in an increasing methanol yield and, after surpassing an ideal M/Cu (M = Pt or Pd) ratio, the yield decrease.

HAADF-STEM micrographs show small dispersed clusters for all PtCu-MOR_8.5_ materials after activation at 200 °C (Fig. 3 and S7†). The material with the least platinum, PtCu-MOR_8.5_(0.02) showed larger particles (*ø* = 2.2 nm) compared to PtCu-MOR_8.5_(0.08) (*ø* = 1.1 nm), PtCu-MOR_8.5_(0.20) (*ø* = 0.65 nm) and PtCu-MOR_8.5_(0.35) (*ø* = 0.80 nm). However, if a sample undergoes 450 °C activation, agglomerated particles were observed, which can be responsible for the low activity after high temperature activation (Fig. S12†). Further, powder X-ray diffraction (PXRD) was carried out for a series of Cu-MOR that was impregnated with different amounts Pt(NH_3_)_4_(NO_3_)_2_ and dried at 110 °C. The materials mostly showed an additional reflection at about 2*θ* = 13° and further additional peak in case of H_2_PtCl_6_, which consequently comes from a platinum-containing phase (Fig. S8†). These additional reflections significantly decreased in intensity during activation in oxygen for 2 h at 200 °C and did not decrease significantly within four further hours of activation at 200 °C, indicating that these corresponding precursors are converted to the active species. After a cycle including 200 °C activation, methane reaction and extraction with liquid water, the reflections disappeared in all cases (Fig. S10†).

**Fig. 3:**
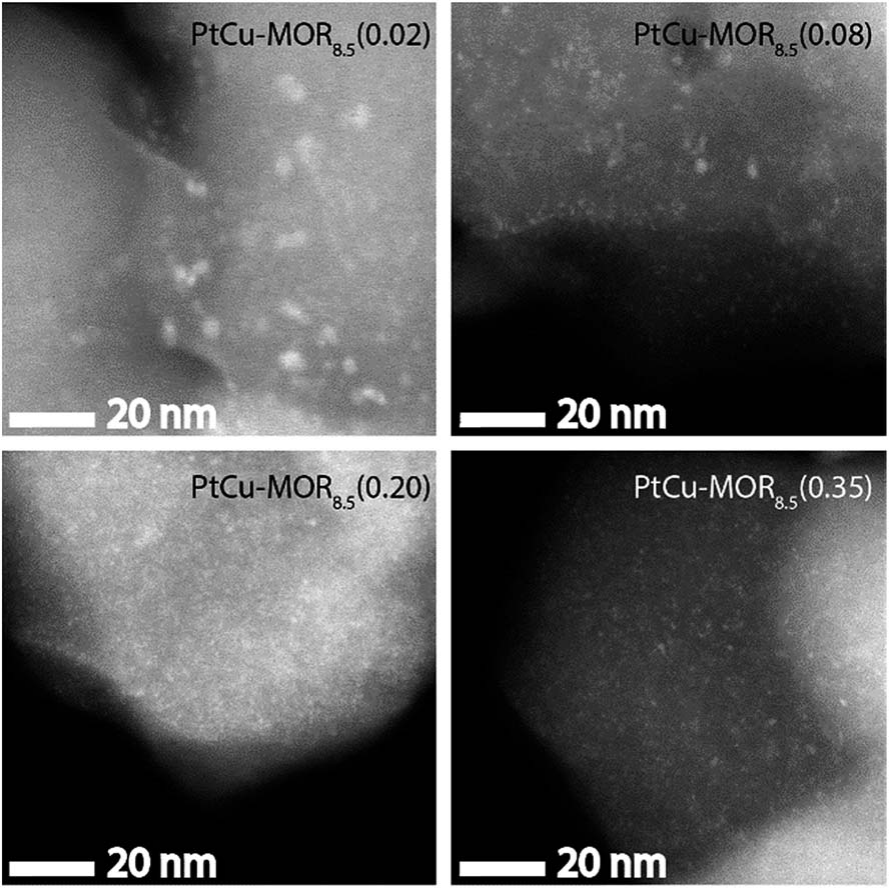
HAADF-STEM micrographs of the different PtCu-MOR_8.5_ materials after activation in a flow of oxygen (50 mL min^–1^) at 200 °C for 13 h. Scale bar is 20 nm.

Copper oxide clusters are the active species for the stepwise isothermal conversion of methane to methanol at elevated methane pressures.^36^ PtCu- and PdCu-MOR, however, show higher activity than Cu-MOR, which must in turn be related to the nature of the bimetallic Cu-oxo clusters. On the one hand there are clusters, some in the sub-nm range, which allow for a high fraction of active surface sites. On the other hand, the introduction of platinum is reported to facilitate the reduction of copper, as it is shown by lower reduction temperatures in temperature programmed reduction for PtCu-MOR compared to the monometallic compounds.^43^ Further, the introduction of platinum into bimetallic PtCu/SiO_2_ catalysts was reported to increase dispersion and reducibility of the copper species.^44^

Thus, besides from the availability of many surface atoms, the reducibility seems to be of key importance for activity under isothermal conditions. This could already be seen by the positive effect of higher methane pressures, which relate to higher reducing power.^36^ For the case of bimetallic oxidized PtCu- and PdCu-MOR it is the reducibility of the bimetallic Cu-oxo clusters that is responsible for the increased activity under isothermal conditions. High temperature activation (450 °C) resulted in larger agglomerates for bimetallic oxide materials, while Cu-MOR gave high yields. Under these conditions, Cu-zeolites form highly active species that easily react even with low pressures of methane. If high pressures of methane are introduced, these species might easily be reduced to clusters that cannot retain the methoxy precursor.

Next, the recyclability of the materials was tested. Extraction in liquid water of PtCu-MOR_6_ with Pt/Cu_theory_ > 0.32 showed visible Pt-leaching (Fig. S13†). To prevent leaching of Pt to the liquid phase, we investigated successive cycles of activation, reaction with methane and steam extraction using PtCu-MOR_6_(0.15) and also PdCu-MOR_6_(0.3) over four cycles (Fig. 4). A stable yield of 6.9–7.4 μmol g^–1^ for PtCu-MOR_6_(0.15) and 6.5–7.8 μmol g^–1^ for PdCu-MOR (0.3) was achieved. These yields are similar to those obtained by off-line extraction in liquid water, which are 8.6 μmol g^–1^ and 7.2 μmol g^–1^, respectively. The materials are recyclable for at least four cycles. Active PtCu-MOR_6_(0.15) after four successive cycles at 200 °C, however, showed rather small particles with a mean particle diameter of 3.2 nm without large agglomerated nanoparticles (Fig. S14†).

**Fig. 4:**
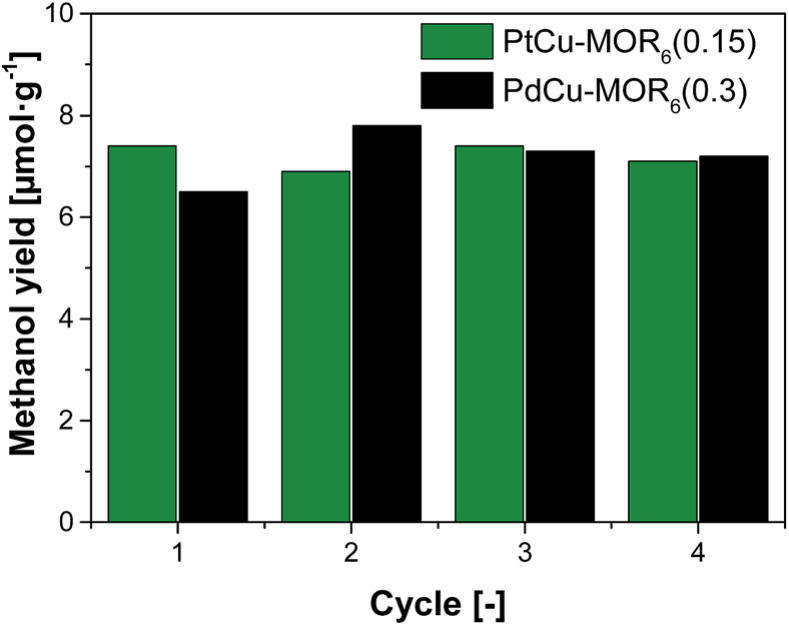
Methanol yield after successive cycles of activation in oxygen and reaction in methane and extraction using steam at 200 °C (isothermal) at ambient methane pressure for PtCu-MOR_6_(0.15) and PdCu-MOR_6_(0.3).

Conclusively, we have pioneered the use of PtCu-MOR and PdCu-MOR materials for the stepwise isothermal conversion of methane to methanol. Upon platinum- or palladium-introduction into the system, the activity after activation and reaction at 200 °C significantly increased. In contrast to all prior approaches for the stepwise methane to methanol conversion, these materials are more active after activation at low temperature than after high temperature activation. Although promising results were obtained, the best yields of Cu-zeolites after high temperature activation are still to be reached under isothermal conditions. The character of the bimetallic Cu-oxo clusters facilitate reduction of the Cu-oxides, which enables reactivity of less activated sites. These bimetallic oxide materials allow for higher activity than other reported materials under isothermal conditions at ambient pressures. We strongly believe that this study will stimulate new research and eventually such bimetallic oxide materials can be a further step to finding the holy grail of catalysis.

## Conflicts of interest

There are no conflicts to declare.

## Supplementary Material

Supplementary information
