# Supplementary material for: Increasing the activity of copper exchanged mordenite in the direct isothermal conversion of methane to methanol by Pt and Pd doping
Source: Chem Sci. 2018 Oct 3;10(1):167–71. doi: 10.1039/c8sc02795a (PMC6330690; doi:10.1039/c8sc02795a)
Supplement: Supplementary file 1 [file SC-010-C8SC02795A-s001.pdf]

Electronic Supplementary Material (ESI) for Chemical Science.
This journal is © The Royal Society of Chemistry 2018

Supporting Information

Increasing the activity of copper exchanged mordenite in the direct isothermal conversion of methane to methanol by Pt and Pd doping

P. Tomkins,[a,b] A. Mansouri,[a,b] V. Sushkevich,[a] L. I. van der Wal,[a,c] S. E. Bozbag,[a,b] F. Krumeich,[b] M. Ranocchiari,[a] J. A. van Bokhoven[a,b]*

[a] Paul Scherrer Institut, CH-5232 Villigen, Switzerland.
[b] ETH Zurich, Wolfgang-Pauli-Strasse 10, CH-8093 Zurich, Switzerland.
[c] Utrecht University, Universiteitsweg 99, 3584 CG Utrecht, The Netherlands

Experimental datails

Materials

Cu-MOR

Copper-exchanged zeolites were synthesized in a 0.01 M solution of $Cu(OAc)_2$ $3H_2O$ (Sigma Aldrich) for Cu-$MOR_{8.5}$ (Zeocat FM-8, Zeochem AG, Si/Al=8.5) and $Cu(NO_3)_2\cdot 3H_2O$ for (Pt-)Cu-MOR_6 and $Cu(OAc)_2\cdot H_2O$ for (Pd-)Cu-MOR_6 in DI water to which 1 g of NaMOR (Zeochem Zeoflair 800, Zeochem AG, Si/Al=6) was added per 78 mL of solution. The suspension was stirred at a rate of 625 rpm with a magnetic stirrer bar for 24 h at room temperature and then filtered through a glass suction filter (Por.4). The unwashed filter cake was suspended in an identical 0.01M copper solution and stirred for another 24 h. The second step was repeated once more, and the resulting filter cake was washed with 1 L DI water and dried for 10 h at 110 °C. Copper mordenite with a lower copper was been synthesized in an analogous way, but only carrying out the ion exchange once with a 0.05 M solution of copper acetate. This sample with a lower copper loading was employed for the preparation of PtCu $MOR_{8.5}$(0.35).

Preparation of PtCu-$MOR_{8.5}$(x)

A platinum containing solution was prepared by dissolving $Pt(NH_3)_4(NO_3)_2$ in water (4.7 mL per gram Cu-MOR). Cu-MOR (usually 4 g) was added to the solution, and the suspension stirred for 60 min. The solvent was removed while stirring at 85 °C. The resulting solid was dried for 10 h at 110 °C. The amount of $Pt(NH_3)_4(NO_3)_2$ employed was calculated to give X=0.2 wt%, 1 wt% or 2.6 wt% Pt in the dried catalyst.

Preparation of Pd/PtCu-MOR_6(x)

Pt/Cu mordenite materials were synthesized via impregnation of the as prepared Cu-MOR material with different concentrations of an aqueous $H_2PtCl_6\cdot$6H2O solution for the preparation of PtCu-MOR_6 or $PdCl_2$ dissolved in 30 % HCl for the preparation of PdCu-MOR_6 *via* the incipient wetness impregnation method. The amount of $H_2PtCl_6\cdot$6H2O was calculated according to the desired Pt/Cu ratio mentioned in the article. Approximately 2 g of Cu-MOR was weighed. Then a volume corresponding to the pore volume was added drop-wise, while stirring the material in order to distribute the precursor solution homogeneously. After adding the desired amount of solution, the Pt/Cu mordenites were dried at 110 °C for 10 h.

Catalytic testing

Ambient pressure activity testing

The materials were sieved into a fraction of 250-500µm. Subsequently the material (approximately 0.5 g) was loaded into a stainless steel reactor (D=8 mm, L=60 cm). Activation of the samples occurred under a pure O_2 flow of 50 mL/min. The temperature increase was 10 °C/min, until the desired temperature of 200 °C was reached. After 2 hours of activation at 200 °C under 50 mL/min O_2 flow, the reactor was purged with a 30 mL/min He flow for 30 minutes. After the purging step, a CH4 flow of 3600 ml g^{-1} h^{-1} was led through the reactor. This flow corresponded to 30 mL/min for 0.5 gram of material. The reaction took place for30 minutes, after which the flow was changed to 30 mL/min He and the reactor was cooled down to room temperature. Methanol was extracted off-line by adding 2 mL of MilliQ water at room temperature and stirring this suspension for 1 hour. This extraction procedure was performed twice per tested material. A complete reaction cycle from reaction with methane, extraction with steam followed by regeneration in oxygen was monitored with mass spectrometer connected to the reactor outlet to identify the reaction byproducts. For a typical regeneration, the spent material after extraction of methanol was exposed to helium for 30 min and then pure oxygen at the constant temperature of 200 °C for 2 h. Afterwards, the reacor was purged with helium before introducing methane. The PtCu-$MOR_{8.5}$(x) materials were tested using the same procedure, yet at a heating rate of 1 °C/min and activation for approximately 16 h. Higher activation temperatures were kept for 4 h.

High pressure activity testing

High pressure activity testing was performed in a 60 mL stainless steel autoclave of the brand premex reactor ag, made of 1.4980 stainless steel. The material (0.5 g, particle size 250-500 µm) was loaded to the reactor and subsequently activated under a pure O_2 flow of 70 mL/min. The temperature was increased with 10 °C/min. Once the desired activation temperature of 200 °C was reached, the reactor was kept at this temperature for 2 hours. The reactor was purged with helium (10 times at 3 bar), resulting in a small temperature increase of approximately 10 °C. Once the temperature was stable again, the reactor was purged with methane (10 times at 3 bar). The gas flow was stopped and the pressure was increased to the desired pressure (6 bar or 36 bar), at which the reaction lasted for 30 minutes. The reactor was then cooled down to room temperature under a helium flow, after which methanol was extracted off-line by adding 2 mL of MilliQ water at room temperature and stirring this suspension for 1 hour. This extraction procedure was performed twice per tested material.

Gas chromatographic analysis of the extracted methanol solution

Analysis of the off-line extraction solution was performed with gas chromatography, carried out on an Agilent 6890 GC (Restek RTX® - 5 capillary column, 3m, internal diameter of 0.25mm) equipped with an FID detector. Pure helium was used as carrier gas. The oven was kept at 40 °C. After injection with 0.4 µL of the sample, the oven temperature was increased with 10 °C/min up to a maximum temperature of 100 °C, at which the temperature was kept for 1 min after which the oven was cooled down to 40 °C for the following injection. Each sample was injected 5 times, after which the average amount of methanol was determined over the 5 injections. Approximately 1 µmol Acetonitrile per mL solution was used as external standard.

Characterization

Powder X-ray diffraction

Powder XRD measurements were performed on a Bruker D8 Advance diffractometer with an incorporated Lynxeye XEdetector. Cu Ka radiation with a wavelength of 1.5418 Å (Bragg-Brentano geometry) was used for the measurements. The long fine focus was operated at 40kV and 40 mA. For zeolites the diffractograms were recorded from 5 °to 70 °, with a step size of 0.02 and time per step 67.2 s.

Atomic absorption spectroscopy

The composition of the prepared zeolite based materials was measured with Atomic Absorption Spectroscopy (AAS) with an Agilent SpectrAA 220FS device. Approximately 30 mg of the sample was dissolved in 2 mL hydrofluoric acid. After digesting for approximately 30 minutes, 3 mL of of nitric acid (2.5 M) was added. This mixture was left to digest the material overnight, after which the sample was diluted up to 50 mL.

Transmission electron microscopy (TEM)

Scanning Transmission electron microscopy (STEM) Samples of the activated material were prepared by suspending the catalyst in ethanol and by putting some droplets onto a holey carbon film supported on a Mo TEM grid. After drying, the sample was investigated on an aberration-corrected STEM (HD2700CS microscope (Hitachi, CFEG, 200 kV)). Images were recorded with a high-angle annular dark field detector (HAADF) resulting in Z contrast (atomic-number contrast). A energy-dispersive spectrometer attached to this microscope allows performing elemental analysis of nm-sized areas.

X-ray spectroscopy (XPS)

XPS measurements were performed with a VG ESCALAB 220iXL spectrometer (Thermo Fisher Scientific) using focused monochromatized Al Kα radiation (hν = 1486.6 eV). Ag $3d_{5/2}$ was used to calibrate the spectrometer using a binding energy of 368.3 eV. The pressure in the analysis chamber was around 2×10^{-9} mbar at room temperature, and the analyzed areas of the samples were about 500 μm^2 (power of 150 W). The spectra were recorded in a constant analyzer energy mode at pass energy of 30 eV for high-resolution acquisition of core level spectra. The materials were treated ex situ at oxygen activation conditions, and then transferred into the XPS cell. Cu 2p, Pt 4d, and Pd 3d core levels peaks were used to investigate the relative concentrations of copper, platinum, and palladium species, respectively. Due to the charging of the materials during measurements, we calibrated the binding energy scale using Si 2p as a reference (103.4 eV). The Pd 3d, Pt 4d, and Cu 2p photoemission lines were deconvoluted into chemical-shifted components (after Shirley background removal) using Doniach–Sunjic shaped peaks.[1]

Additional tables and figures

Table S1. Copper and platinum contents as determined by AAS and BET surface area of selected materials based on Cu-MOR with Si/Al=8.5.

Sample name	Cu-content [wt%]	Pt-content [wt%]	Theoretical Pt/Cu [mol/mol]	Measured Pt/Cu [mol/mol]	Specific surface area (BET) [$m^2 \cdot g^{-1}$]
CuPt-$MOR_{8.5}$(0.02)	4.05	0.08	0.02	0.006	370
CuPt-$MOR_{8.5}$(0.08)	3.95	0.17	0.08	0.014	312
CuPt-$MOR_{8.5}$(0.20)	3.77	0.34	0.20	0.029	155
CuPt-$MOR_{8.5}$(0.35)	≤2.4[a]	≤2.6[a]	0.35	-	-

[a] Calculated based on metals employed in the synthesis of the material

Table S 2. Elemental composition of the series of Cu(Pt)-MOR_6 materials determined by AAS.

Sample name	Cu/Al ratio [mol/mol]	Pt/Cu [mol/mol]	Theoretical Pt/Cu [mol/mol]
Cu-MOR_6	0.31	-	-
CuPt-MOR_6(0.12)	0.27	0.07	0.12
CuPt-MOR_6(0.21)	0.32	0.07	0.21
CuPt-MOR_6(0.32)	0.34	0.11	0.32
CuPt-MOR_6(0.55)	0.35	0.18	0.55
CuPt-MOR_6(1.11)	0.35	0.30	1.11

Table S 3. The surface concentrations of bimetallic materials calculated based on the XPS data. The materials were treated with atmospheric oxygen at 200 °C.

Sample name	Cu/Pt surface conc.
CuPt-$MOR_{8.5}$(0.15)	0.5
CuPd-$MOR_{8.5}$(0.3)	0.7

Table S 4. Methanol yield of parent and Pd-doped Cu-MOR_6 after 450 ° activation.

Sample name	Methanol yield [µmol·g^{-1}]
Cu-MOR_6	27
CuPd-MOR_6(1.0)	20

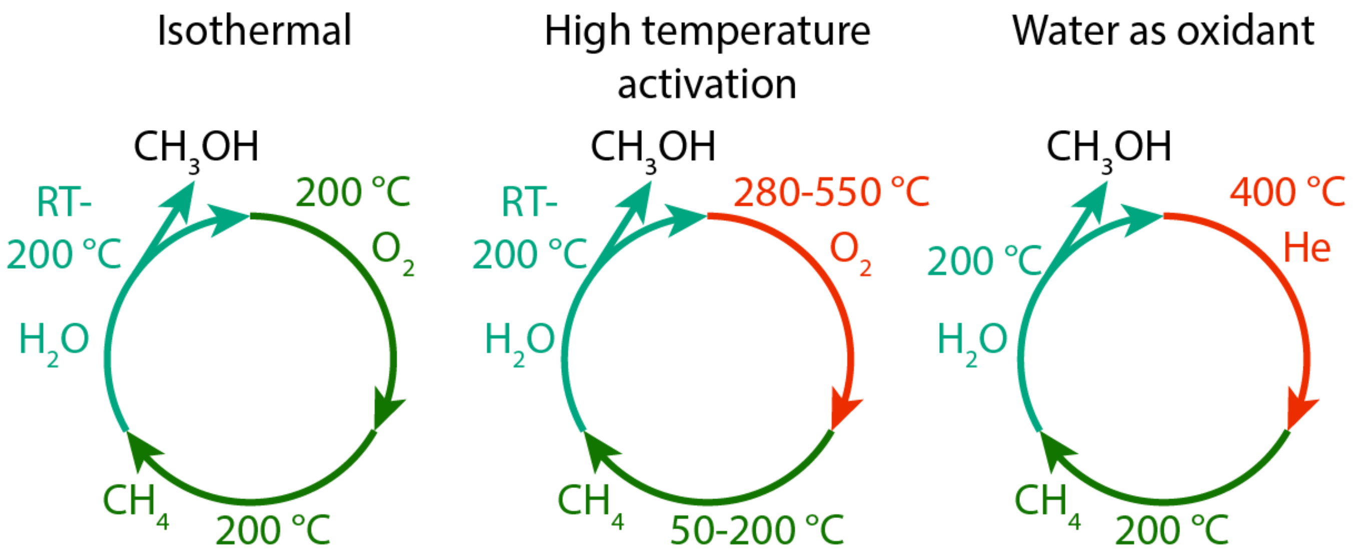

Figure S 1. Representative image for the different modes for the stepped methane to methanol conversion.

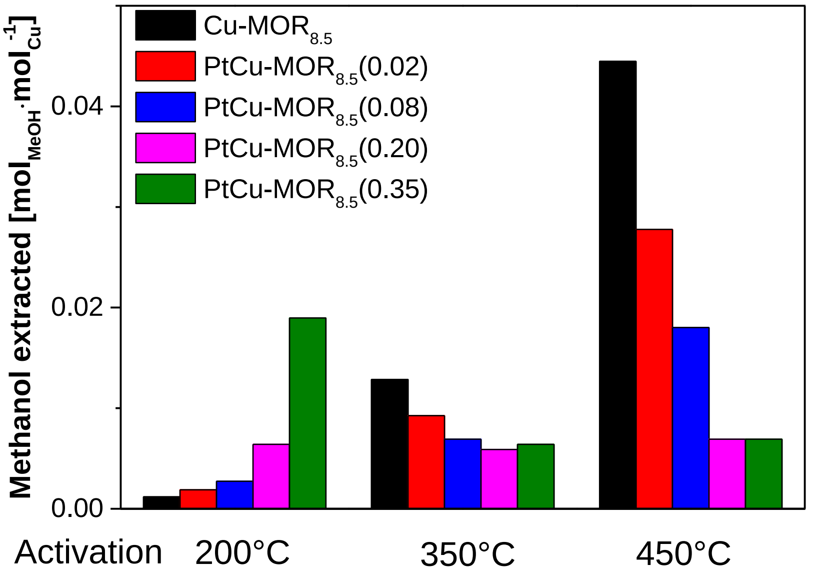

Figure S 2. Methanol yields of different PtCu-MOR$_{8.5}$(Pt/Cu$_{theory}$) materials prepared from Cu-MOR (Si/Al=8.5, 4.3 wt% Cu; ≤2.4 wt% for PtCu-MOR$_{8.5}$(0.35)) after activation at different temperatures normalized to the amount of copper in the sample.

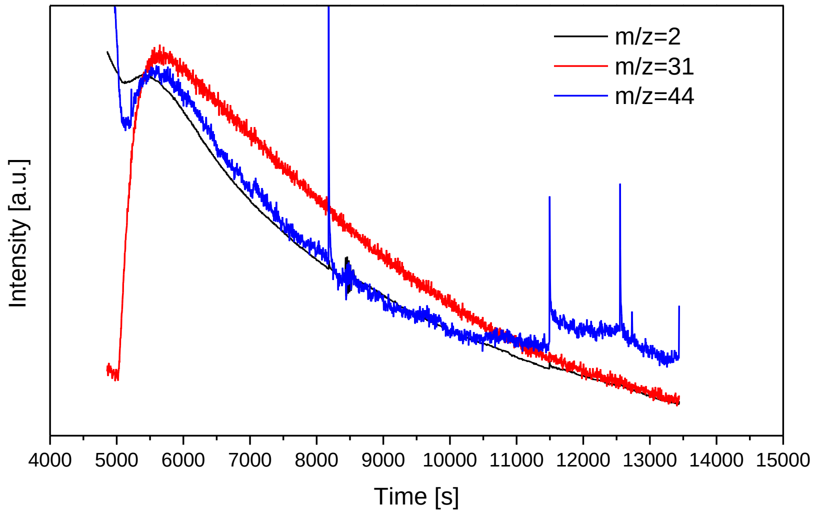

Figure S 3. MS traces corresponding to He (m/z=2), MeOH (m/z=31) and CO_2 (m/z=44) during the extraction using water vapour in helium after an isothermal activation in oxygen and reaction with methane at 200 °C using Cu-MOR$_6$. It is clearly evident that only methanol is extracted.

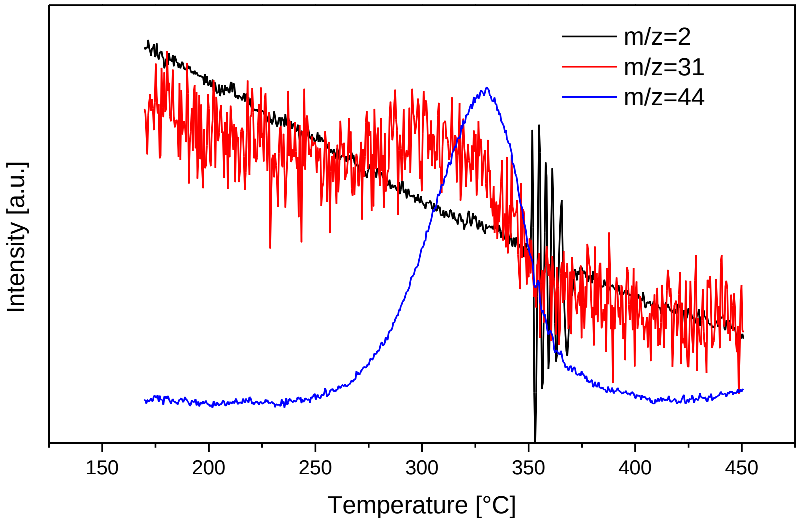

Figure S 4. MS traces corresponding to He (m/z=2), MeOH (m/z=31) and CO_2 (m/z=44) while increasing the temperature in a flow of oxygen after an isothermal activation in oxygen, reaction with methane and extraction of methanol using water vapour in helium at 200 °C using Cu-MOR$_6$. It can be seen that there are strongly adsorbed carbon-species that were not extracted during steam treatment with methanol.

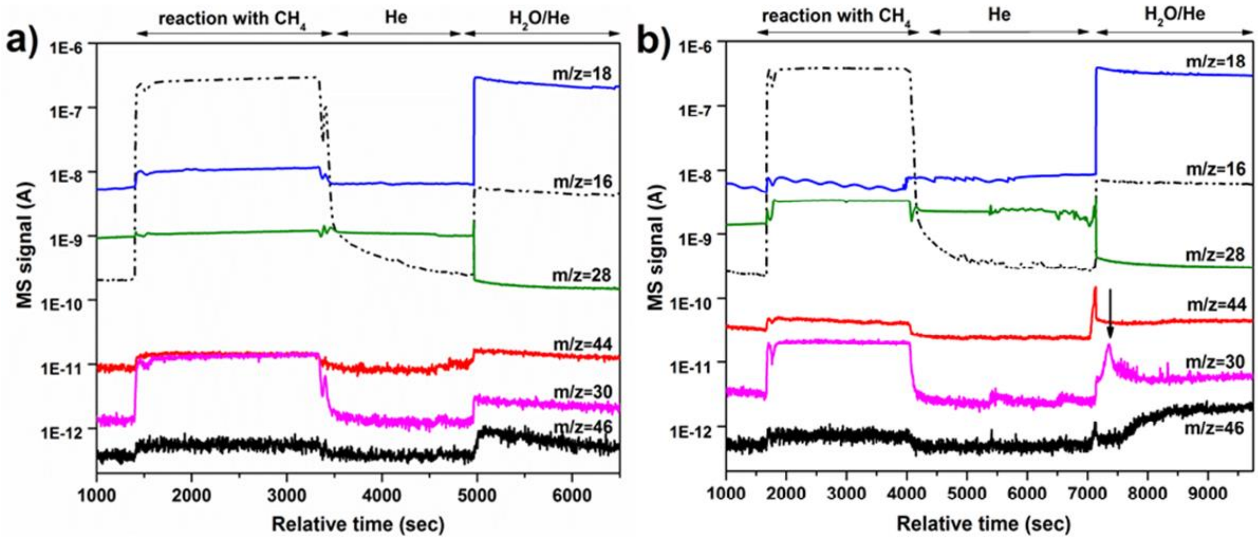

Figure S 5. In situ mass spectrometry analyses of (a) PtCu-MOR(0.15) and (b) PdCu-MOR(0.3) during methane treatment and methanol extraction with water vapor both at 200 °C. The mass of (m/z=16) corresponds to methane, (m/z=18) water, (m/z=28) carbon monoxide, (m/z=30) Formaldehyde, (m/z=44) carbon dioxide and (m/z=46) dimethyl ether. In case of PdCu-MOR (Fig. b), a peak corresponding to formaldehyde formation is observed upon steaming the material to extract methanol.

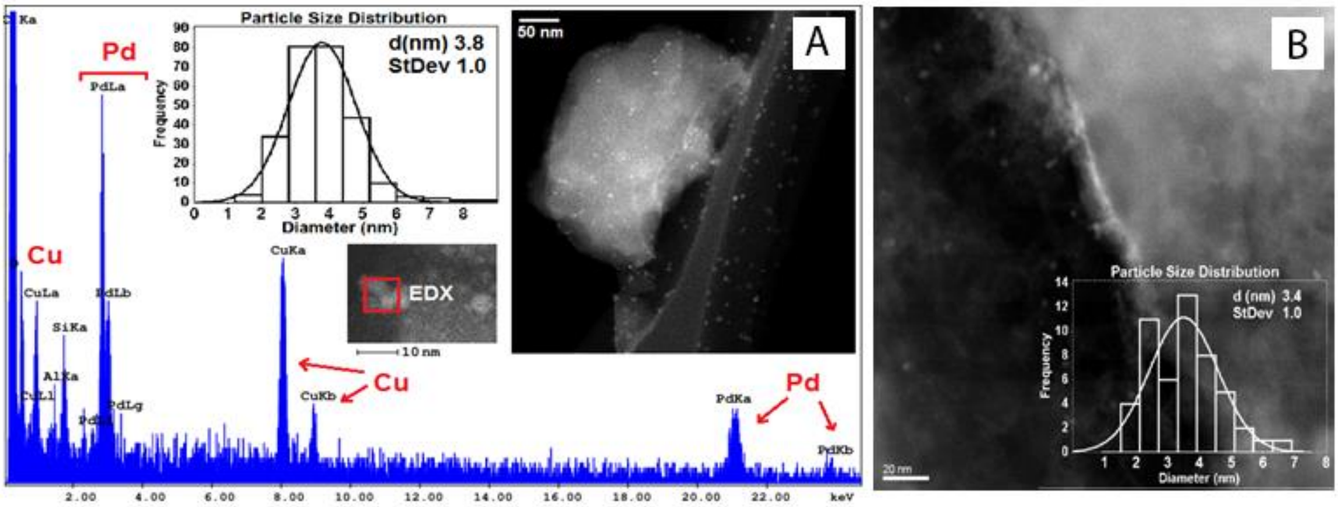

Figure S 6. HAADF-STEM images of PdCu-MOR(0.3) after oxygen activation and methane treatment at 200 °C with a local EDX analysis (A) and PdCu-MOR(0.3) after four isothermal cyclic reactions at 200 °C (B). Size distribution histograms are determined by accounting more than 300 particles.

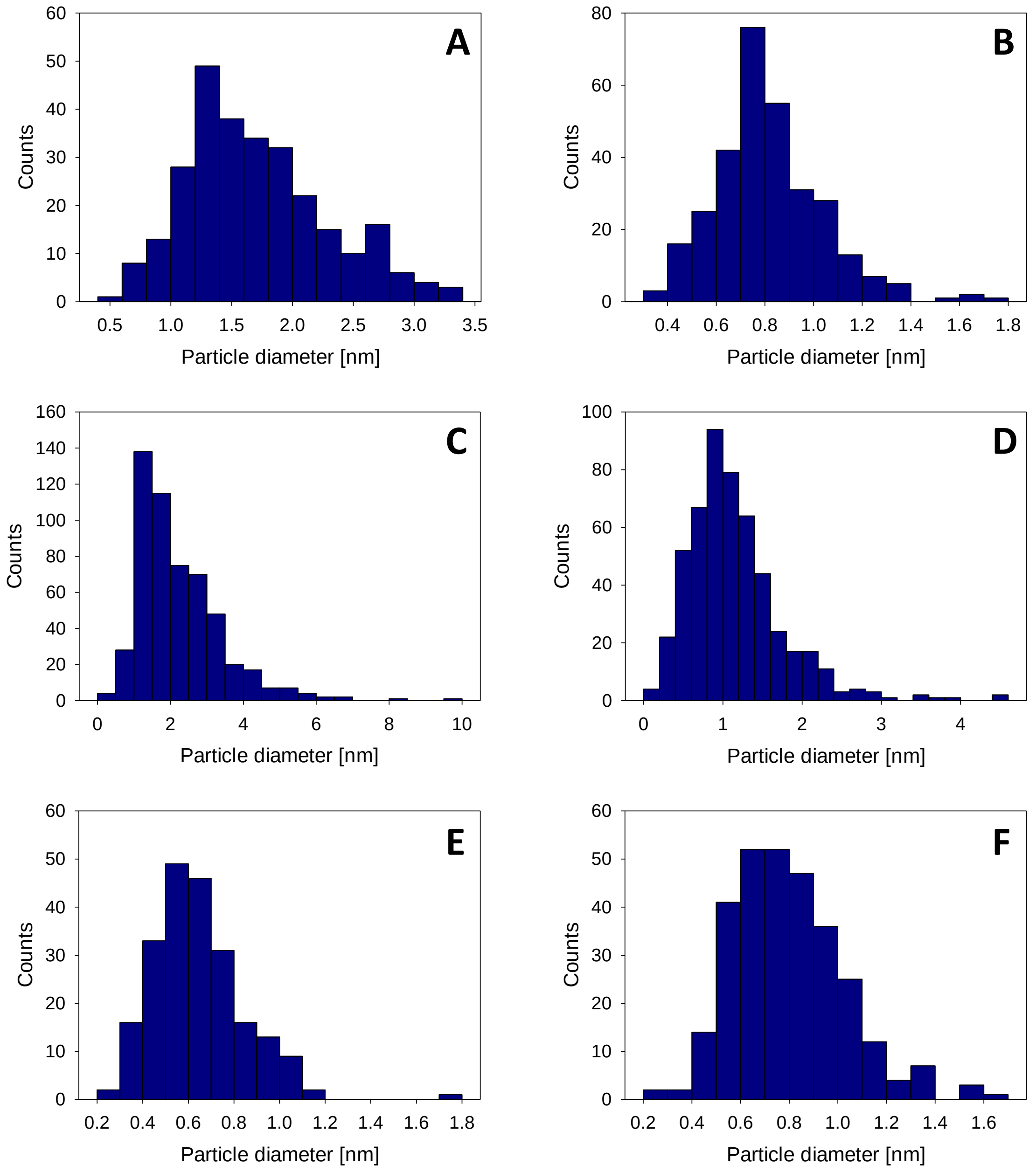

Figure S 7. Histograms for (A) Cu-MOR$_{8.5}$, (B) Pt-MOR$_{8.5}$, (C) PtCu-MOR$_{8.5}$(0.02), (D) PtCu-MOR$_{8.5}$(0.08), (E) PtCu-MOR$_{8.5}$(0.20) and PtCu-MOR$_{8.5}$(0.35) after activation in a flow of oxygen (50 mL·min^{-1}) at 200 °C for 13 h.

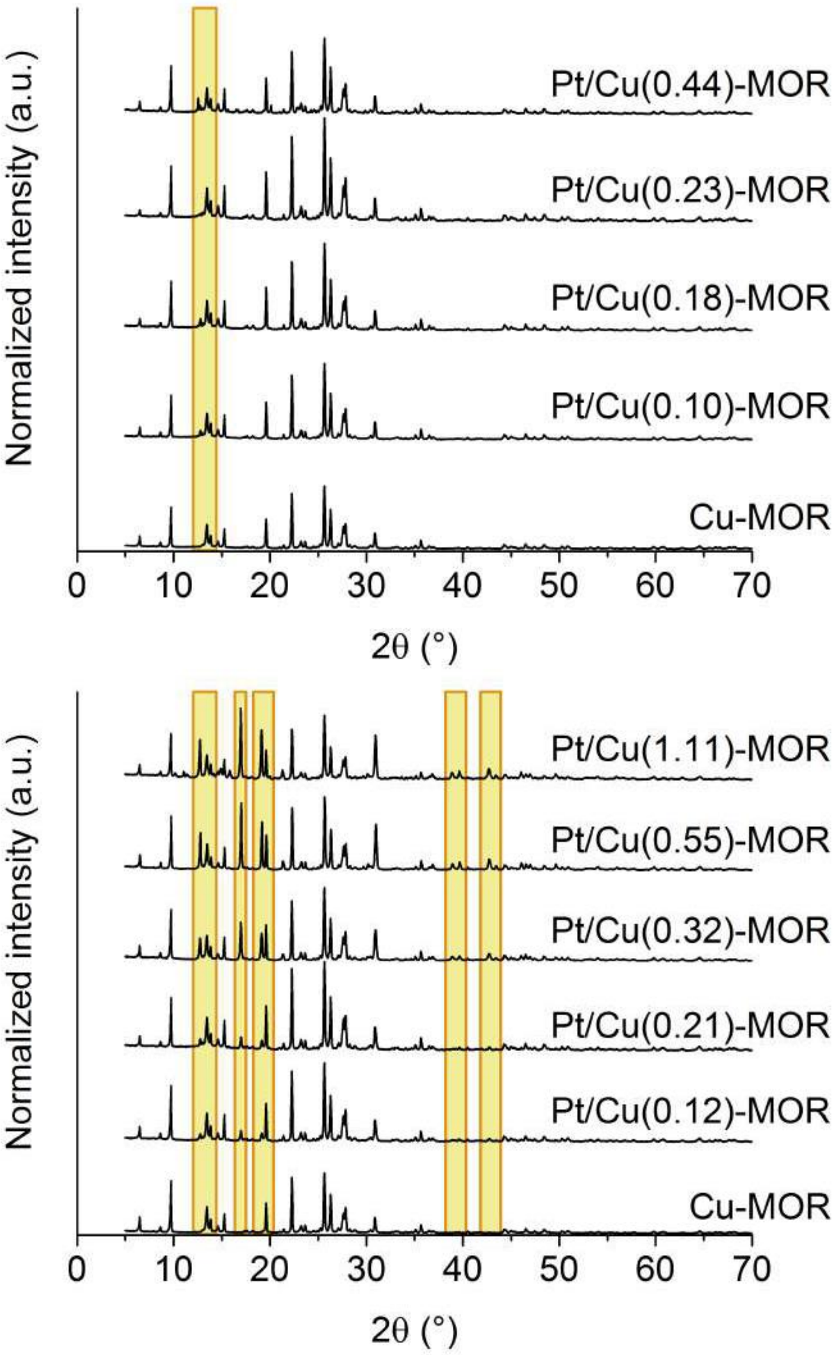

Figure S 8. PXRD pattern of PtCu-MOR$_6$(Pt/Cu$_{theory}$) after incipient wetness impregnation using $Pt(NH_3)_4(NO_3)_2$ (top) and H_2PtCl_6 (bottom) as platinum precursor. Yellow rectangles indicate where diffraction reflections appeared after introducing the platinum precursor.

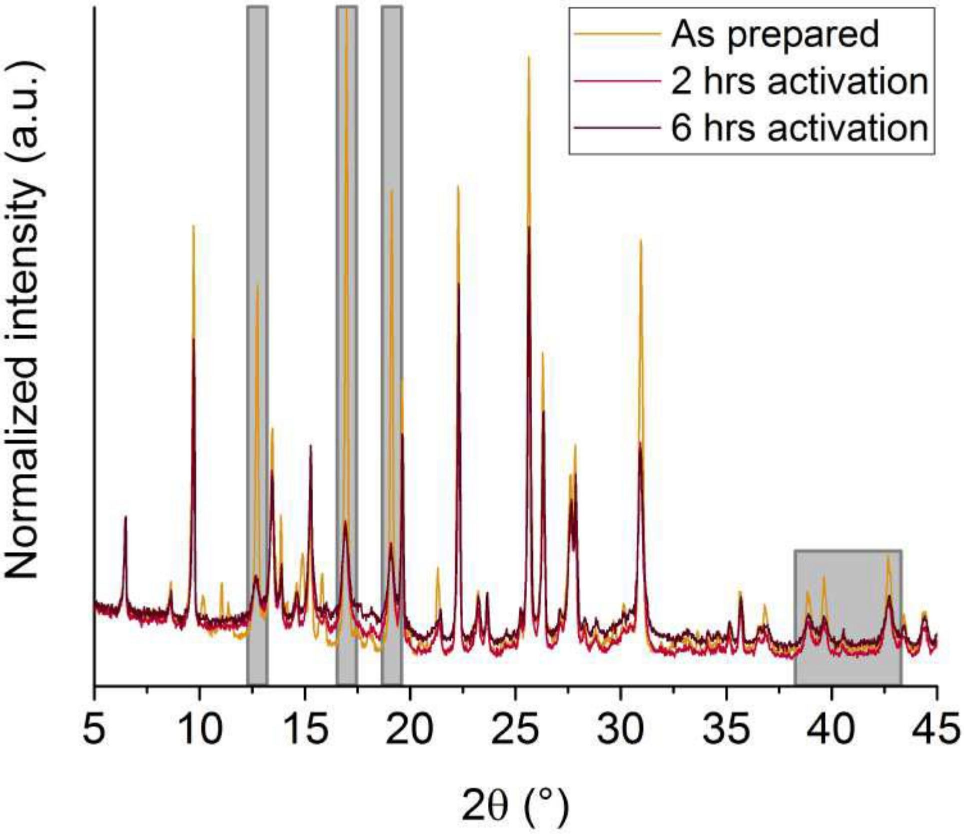

Figure S 9. PXRD pattern of PtCu-MOR$_6$(1.11) after incipient wetness impregnation using and H_2PtCl_6 before and after activation in oxygen at 200 °C for 2, respective 6 h. Grey rectangles indicate where diffraction reflections appeared after introducing the platinum precursor.

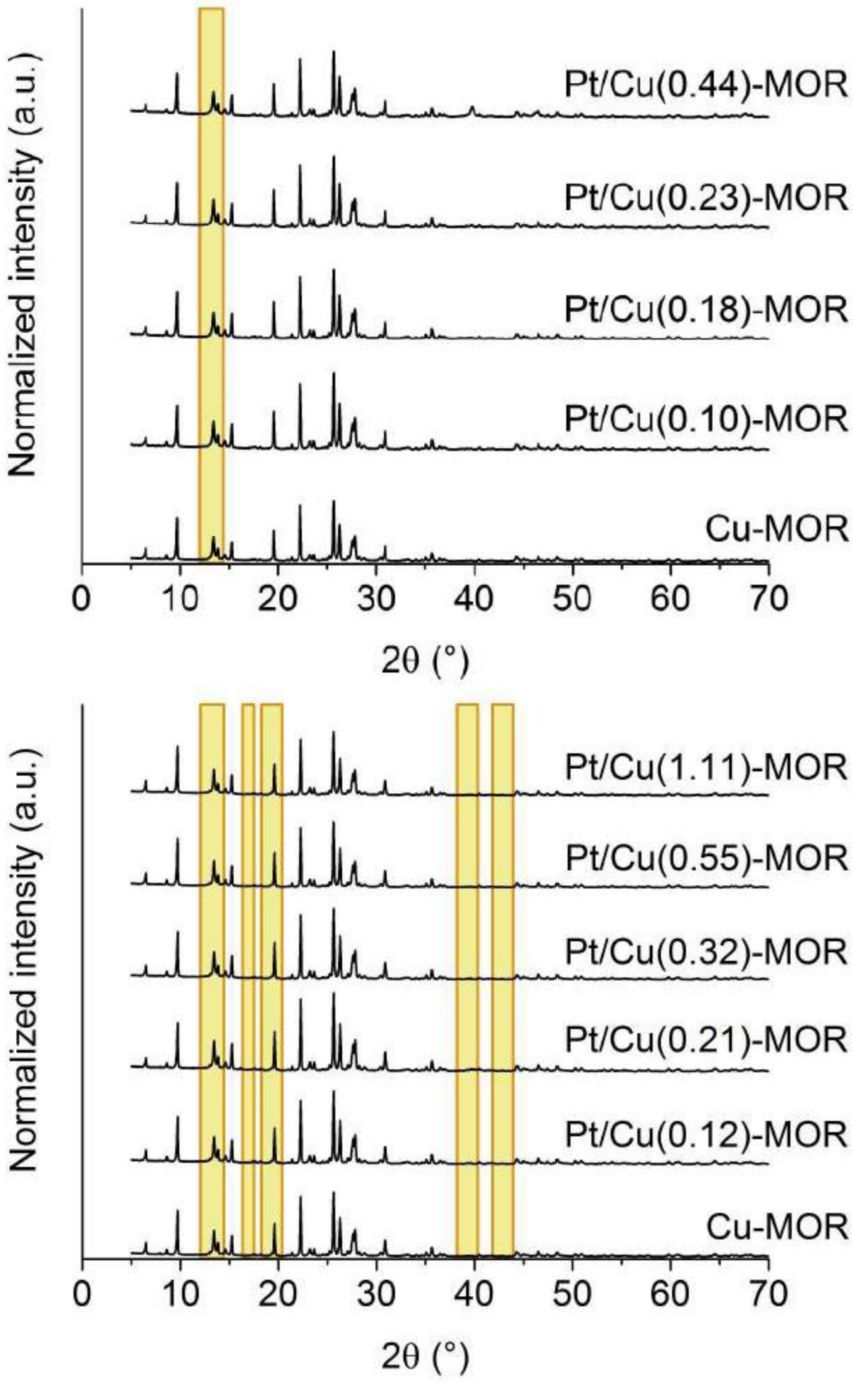

Figure S 10. PXRD pattern of PtCu-MOR$_6$(Pt/Cu$_{theory}$) using $Pt(NH_3)_4(NO_3)_2$ (top) and H_2PtCl_6 (bottom) as platinum precursor after a cycle including activation at 200 °C and reaction with 1 bar methane. Yellow rectangles indicate where diffraction peaks for the platinum precursor were observed before catalysis and disappeared after reaction with methane.

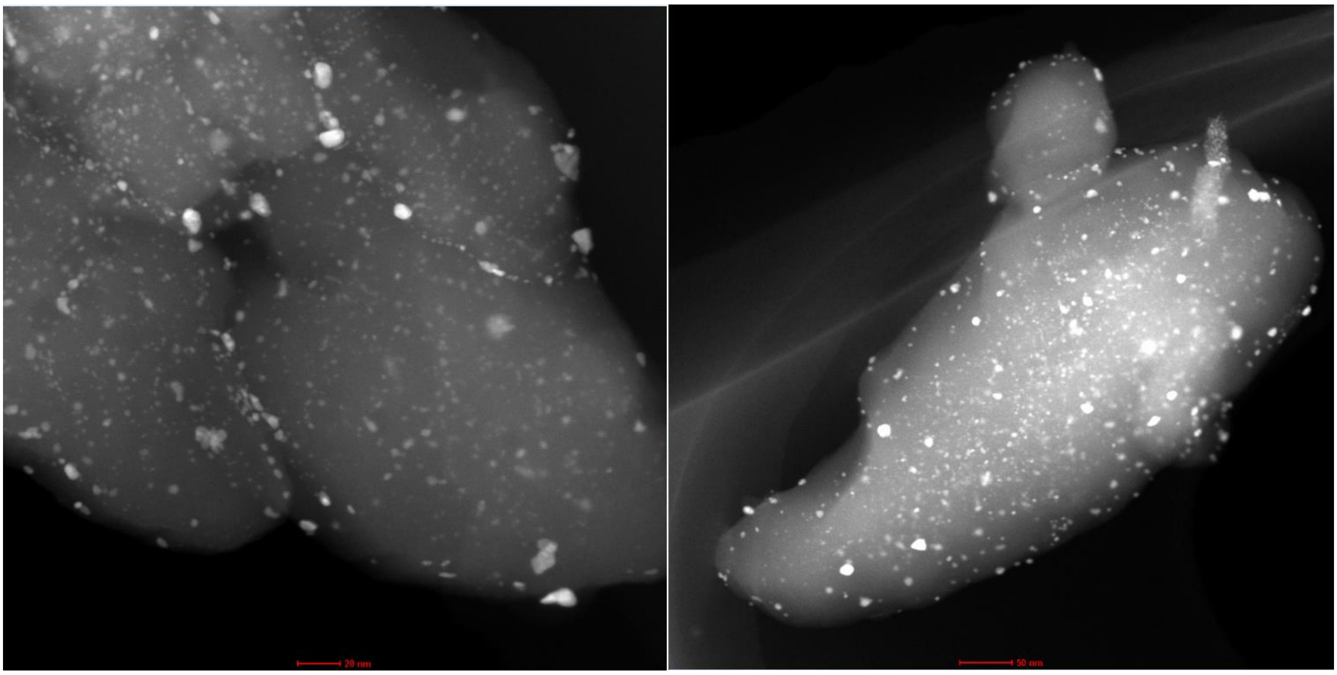

Figure S 11. TEM micrograph of PtCu-MOR$_{8.5}$(0.35) after three successive isothermal cycles at 200 °C and a further cycle with 450 °C including suspending the material in water after each cycle.

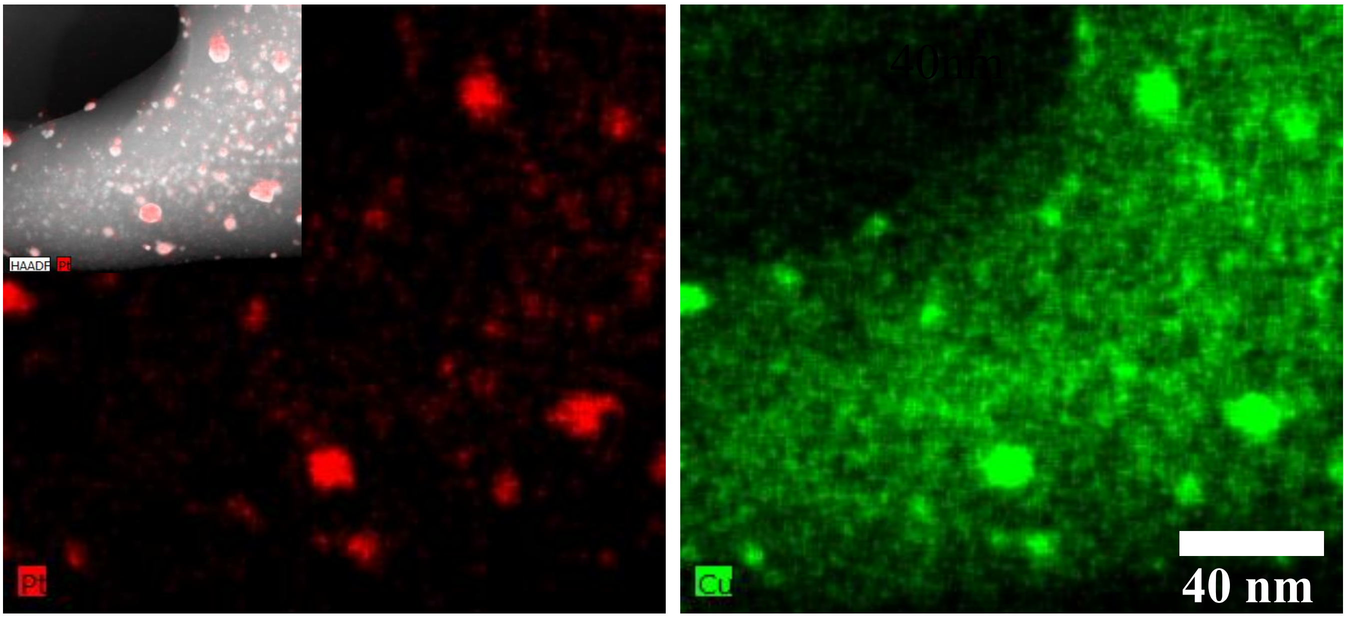

Figure S 12. EDXS mapping of PtCu-MOR$_{8.5}$(0.35) after three successive isothermal cycles at 200 °C and a further cycle with 450 °C including suspending the material in water after each cycle on a copper-grid. It is clearly visible that there are large nanoparticles present. For Cu there is also a contribution from the Cu-grid, yet a higher abundance is visible in the nanoparticles, whereas the contribution of the Cu-grid cannot be higher in these regions. Such large particles were not observed after 5 isothermal cycles at 200 °C.

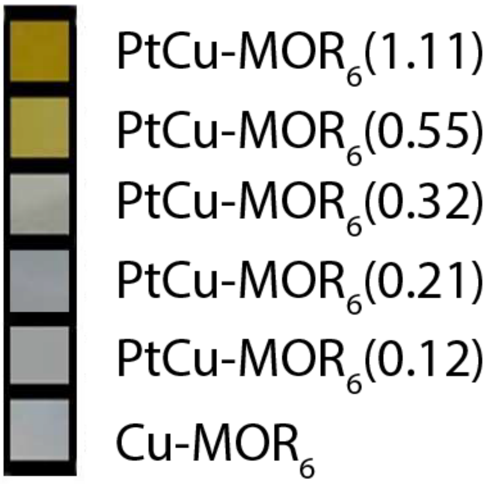

Figure S 13. Colour of the extraction solution after activation in a flow of oxygen and reaction with methane at 200 °C using different PtCu-MOR6(Pt/Cu_{theory}) prepared by incipient wetness impregnation of Cu-MOR_6 with different amounts of H_2PtCl_6.

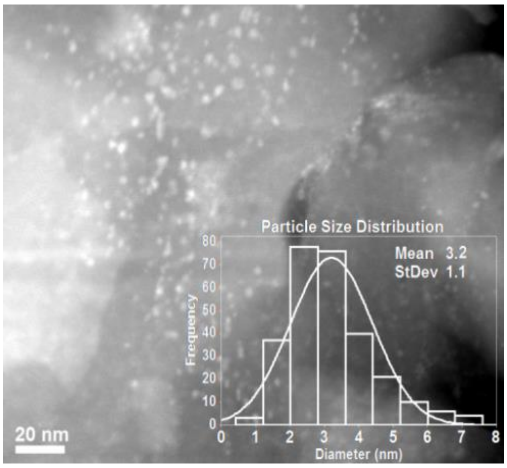

Figure S 14. TEM micrograph of PtCu-MOR_6(0.15) after three successive cycles of activation in oxygen and reaction in methane and extraction using steam at 200 °C (isothermal).

References

1 Doniach, S. and Sunjic, M. *J. Phys. C.* **1970**, *3*, 285–291.
